# Large-scale expansion of γδ T cells and peptide-specific cytotoxic T cells using zoledronate for adoptive immunotherapy

**DOI:** 10.3892/ijo.2014.2634

**Published:** 2014-09-03

**Authors:** TOSHIAKI YOSHIKAWA, MASASHI TAKAHARA, MAI TOMIYAMA, MIE NIEDA, RYUJI MAEKAWA, TETSUYA NAKATSURA

**Affiliations:** 1 Division of Cancer Immunotherapy, Exploratory Oncology Research and Clinical Trial Center, National Cancer Center, Kashiwa 277-8577, Japan; 2 Medinet Medical Institute, Setagaya-ku, Tokyo 158-0096, Japan

**Keywords:** adoptive cell transfer, cytotoxic T lymphocyte, γδ T cell, glypican-3, hepatocellular carcinoma

## Abstract

Specific cellular immunotherapy for cancer requires efficient generation and expansion of cytotoxic T lymphocytes (CTLs) that recognize tumor-associated antigens. However, it is difficult to isolate and expand functionally active T-cells *ex vivo*. In this study, we investigated the efficacy of a new method to induce expansion of antigen-specific CTLs for adoptive immunotherapy. We used tumor-associated antigen glypican-3 (GPC3)-derived peptide and cytomegalovirus (CMV)-derived peptide as antigens. Treatment of human peripheral blood mononuclear cells (PBMCs) with zoledronate is a method that enables large-scale γδ T-cell expansion. To induce expansion of γδ T cells and antigen-specific CTLs, the PBMCs of healthy volunteers or patients vaccinated with GPC3 peptide were cultured with both peptide and zoledronate for 14 days. The expansion of γδ T cells and peptide-specific CTLs from a few PBMCs using zoledronate yields cell numbers sufficient for adoptive transfer. The rate of increase of GPC3-specific CTLs was approximately 24- to 170,000-fold. These CD8^+^ cells, including CTLs, showed GPC3-specific cytotoxicity against SK-Hep-1/hGPC3 and T2 pulsed with GPC3 peptide, but not against SK-Hep-1/vec and T2 pulsed with human immunodeficiency virus peptide. On the other hand, CD8^−^ cells, including γδ T cells, showed cytotoxicity against SK-Hep-1/hGPC3 and SK-Hep-1/vec, but did not show GPC3 specificity. Furthermore, adoptive cell transfer of CD8^+^ cells, CD8^−^ cells, and total cells after expansion significantly inhibited tumor growth in an NOD/SCID mouse model. This study indicates that simultaneous expansion of γδ T cells and peptide-specific CTLs using zoledronate is useful for adoptive immunotherapy.

## Introduction

Current therapeutic options for cancer treatment, including surgery, radiotherapy and chemotherapy, have made advancements in recent years and the survival rate of patients with cancer has gradually improved. However, these therapies remain far from satisfactory in most cancers (1,2). Therefore, the development of novel treatment modalities, including antigen-specific cancer immunotherapies with peptide vaccines, dendritic cell vaccines and adoptive cell transfer therapies, is critical for the further advancement of effective cancer treatments (3–5).

We found that glypican-3 (GPC3), which is an oncofetal antigen that is overexpressed in human hepatocellular carcinoma (HCC), was shown to be a useful target antigen for immunotherapy in several studies (6–10). Based on results obtained from preclinical studies, we conducted a phase I clinical trial using a GPC3-derived peptide vaccine in 33 patients with advanced HCC. In almost all vaccinated patients, the frequency of GPC3 peptide-specific CTLs increased after vaccination. Furthermore, this was the first study to show that the frequency of peptide-specific CTLs was correlated with overall survival in patients with HCC receiving peptide vaccines (11,12). Although the peptide vaccine is a potentially attractive treatment modality, the antitumor effects of the peptide vaccine alone are not dramatic in patients with advanced HCC. Therefore, the establishment of an innovative strategy to enhance the power of antigen-specific cancer immunotherapy is urgently required.

Cellular immunotherapy of solid and hematopoietic malignancies is regarded as a promising approach to treat relapse after or resistance to conventional treatments. The adoptive transfer of autologous tumor-infiltrating lymphocytes (TILs) results in objective cancer regression in 49 to 72% of patients with metastatic melanoma (13). However, due to the scarcity of TILs, this therapy is only possible for a limited number of patients. It is difficult to isolate and expand functionally active T cells. Development of a new method of CTL expansion may be useful in addressing this problem.

It was recently reported that γδ T cells are attractive mediators of cancer immunotherapy (14). Several clinical studies that included manipulation of γδ T cells by aminobisphosphonate administration or adoptive transfer of γδ T cells were performed (15–17). γδ T cells recognize their targets independently of major histocompatibility complex (MHC)-mediated antigen presentation (18–21). Human γδ T cells kill a vast repertoire of tumor cell lines and primary samples *in vitro*, including leukemia, lymphoma, melanoma, neuroblastoma and multiple types of carcinomas (22–25). In addition, human γδ T cells mediate antibody-dependent cellular cytotoxicity (26,27). On the other hand, activated human γδ T cells produce large amounts of interferon-γ (28,29), a central cytokine in antitumor immune responses. Moreover, it has been reported that zoledronate stimulates proliferation of γδ T cells, which then stimulate CTLs as antigen-presenting cells (APCs) (30–33). Therefore, we have attempted to use γδ T cells as both effector cells and APCs.

We report on the development of a more effective adoptive immunotherapy. We investigated a new method to induce expansion of γδ T cells and peptide-specific CTLs using zoledronate.

## Materials and methods

### Patient samples

Patient blood samples were obtained during the performance of clinical trials at National Cancer Center Hospital East. We carried out two clinical trials involving GPC3-derived peptide vaccine. The phase I trial was carried out among 33 patients with advanced or metastatic HCC from February, 2007 to November, 2009 (11,12). The trial was registered with the University Hospital Medical Information Network Clinical Trials Registry (UMIN-CTR no. 000001395). We subsequently conducted a phase II trial involving the GPC3-derived peptide vaccine as an adjuvant therapy for patients with HCC. Forty patients with initial HCC who had undergone surgery or radiofrequency ablation were enrolled in this phase II trial (UMIN-CTR no. 000002614). These patients were enrolled after providing a written informed consent. Patients were intradermally injected with HLA-A24-restricted GPC3_298-306_ (EYILSLEEL) or HLA-A2-restricted GPC3_144-152_ (FVGEFFTDV) peptide vaccine emulsified with incomplete Freund’s adjuvant (IFA, Montanide ISA-51VG; SEPPIC, Paris, France). This study was approved by the Ethics Committee of the National Cancer Center and conformed to the ethical guidelines of the 1975 Declaration of Helsinki.

### PBMCs

Peripheral blood (30 ml) was obtained from healthy volunteers or patients at the times designated in the protocol (before the first vaccination and 2 weeks after each vaccination). Peripheral blood mononuclear cells (PBMCs) were isolated by standard Ficoll density gradient centrifugation from buffy coats. In this study, we used the remaining PBMCs after immunological monitoring in the clinical trials.

### Cell lines

The human liver cancer cell lines SK-Hep-1 (GPC3^−^, HLA-A*02:01/A*24:02) and SK-Hep-1/hGPC3 (GPC3^+^, HLA-A*02:01/A*24:02) were used as target cells. SK-Hep-1/hGPC3 is an established stable GPC3-expressing cell line transfected with a human GPC3 gene, and SK-Hep-1/vec is an established counterpart cell line in which an empty vector was transfected. T2 (HLA-A*02:01, TAP^−^) and T2A24 (HLA-A*02:01/A*24:02, TAP^−^) cells were pulsed with GPC3 peptide or human immunodeficiency (HIV) peptide at room temperature for 1 h. They were conserved in our laboratory. Cells were cultured at 37°C in RPMI-1640 or DMEM medium (Sigma-Aldrich, St. Louis, MO, USA) supplemented with 10% fetal bovine serum, 100 U/ml penicillin, and 100 μg/ml streptomycin in a humidified atmosphere containing 5% CO_2_.

### Synthetic peptides

The peptides used in this study were as follows: HLA-A*02:01-restricted GPC3_144-152_ (FVGEFFTDV) peptide (American Peptide Company, Sunnyvale, CA), HLA-A*24:02-restricted GPC3_298-306_ (EYILSLEEL) peptide (American Peptide Company), HLA-A*02:01-restricted cytomegalovirus (CMV)_495-503_ (NLVPMVATV) peptide (ProImmune, Rhinebeck, NY, USA), HLA-A*24:02-restricted CMV_341-349_ (QYDPVAALF) peptide (ProImmune), and HLA-A*02:01-restricted HIV_77-85_ (SLYNTYATL) peptide (ProImmune). The peptides were dissolved and diluted in 7% NaHCO_3_ or dimethyl sulfoxide.

### Large-scale expansion using zoledronate

PBMCs were cultured (2×10^6^ cells/well) with zoledronate (5 μM) (Novartis Pharma, Basel, Switzerland) and CMV or GPC3 peptide (10 μM) in AIM-V medium (Gibco) supplemented with 10% human AB serum (Sigma) and recombinant human interleukin (IL)-2 (1,000 IU/ml) (Novartis Pharma) for 14 days. The stimulation procedure was performed at 37°C and 5% CO_2_. Scale-up of cells was performed in accordance with their growth.

### Expansion of peptide-specific CTLs in the absence of zoledronate

To obtain zoledronate-activated γδ T cells, PBMCs were stimulated with zoledronate and IL-2 for 7 days. On day 7, zoledronate-activated γδ T cells were sorted using FACSAria II. CD8^+^ cells and γδ T cells without zoledronate activation were sorted from non-cultured PBMCs using microbeads and FACSAria II, respectively. Dendritic cells (DCs) were induced from CD14^+^ cells using GM-CSF and IL-4. On day 5, DCs were stimulated with TNF-α for 2 days. We used γδ T cells with or without zoledronate activation and TNF-α-stimulated DCs as stimulator cells. Stimulator cells were pulsed with CMV peptide (10 μM) for 1 h at room temperature. After washing out the peptide, stimulator cells were co-cultured for 2 weeks with responder CD8^+^ cells and the addition of IL-2 in the absence of zoledronate. We compared the percentages of CMV peptide-specific CTLs in responder CD8^+^ cells using dextramer assays.

### In vitro stimulation of GPC3 peptide-specific CTL clones

GPC3 peptide-specific CTL clones were previously generated by single cell sorting using a GPC3-dextramer or CD107a antibody. CTL clones were stimulated as described previously (34).

### Dextramer staining and flow cytometry analysis

The PBMCs were stained with CMV, GPC3 or HIV Dextramer-RPE (Immudex, Copenhagen, Denmark) for 10 min at room temperature and with anti-CD8-FITC (ProImmune) or anti-CD8-APC (BioLegend, San Diego, CA), anti-CD45RA-FITC (BD Biosciences, San Jose, CA, USA), and anti-CCR7-PerCP/Cy5.5 (BioLegend) for 20 min at 4°C. To detect γδ T cells, PBMCs were stained with anti-TCR-Vγ9-FITC (Beckman Coulter, Erembodegem, Belgium) and anti-CD3-PC5 (BioLegend) for 20 min at 4°C. γδ T cells, with or without zoledronate activation, and TNF-DCs were stained with anti-HLA-class I-FITC, anti-CD80-FITC, anti-CD83-FITC and anti-CD86-PE (BD Biosciences) antibodies for 20 min at 4°C. Flow cytometry analysis was carried out using FACSCanto II (BD Biosciences).

### Cytotoxicity assay

Cytotoxic activity against target cells was analyzed using the Terascan VPC system (Minerva Tech, Tokyo, Japan) as described previously (34). Target cells were labeled with calcein AM (Dojindo, Kumamoto, Japan) solution for 30 min at 37°C. The labeled cells were then incubated with effector cells for 4 to 6 h. As effector cells, CD8^+^ and CD8^−^ T cells were isolated using human CD8 microbeads (BD Bioscience) from PBMCs stimulated for 14 days. Assays were conducted in duplicate.

### Transfer of effector cells to NOD/SCID mice implanted with the GPC3^+^ or GPC3^−^ cell line

Female NOD/SCID (6–8 weeks old) were purchased from Japan Charles River Laboratories (Yokohama, Japan). All animal procedures were performed according to the guidelines for the Animal Research Committee of the National Cancer Center, Japan. We inoculated SK-Hep-1/hGPC3 or SK-Hep-1/vec cells subcutaneously into the right flank of NOD/SCID mice. We intravenously injected the CD8^+^ cells, CD8^−^ cells, or both, as effector cells. We injected PBS as a negative control. Before adoptive transfer, we examined the percentage of CD8^+^ cells in expanded cells using flow cytometry. The percentage of CD8^+^ cells after expansion was ~25% of all cells. We injected immune cells at this ratio. We injected 5×10^6^ cells per mouse for the CD8^+^-cell-treatment group. We injected 1.5×10^7^ cells per mouse for the CD8^−^ cell treatment group and 2.0×10^7^ cells per mouse for the all cells treatment group. We performed adoptive cell transfer of expanded cells using five mice per group. The tumor volume was monitored and calculated using the following formula: tumor volume (mm^3^) = a × b^2^ × 0.5, where a is the longest diameter, b is the shortest diameter, and 0.5 is a constant to calculate the volume of an ellipsoid.

### Statistical analysis

The correlation between the number of GPC3-specific CTLs and γδ T cells at days 0 and 14 was analyzed using the Spearman’s rank correlation coefficient. Comparisons of tumor volume at the last time point were performed using the Mann-Whitney U test. Differences were considered significant at P<0.05.

## Results

### Zoledronate induces expansion of γδ T cells and peptide-specific CTLs from PBMCs

**Figure 1:**
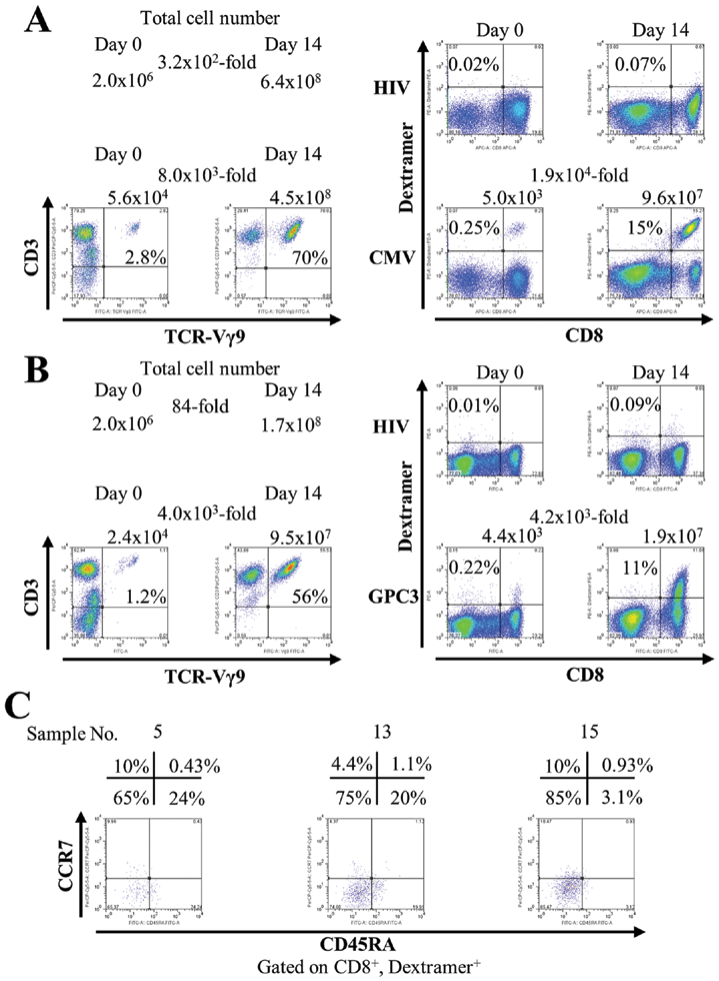
Zoledronate induces expansion of γδ T cells and peptide-specific CTLs. We performed flow cytometry analysis before (day 0) and after (day 14) cell culture. Representative data are shown. (A) PBMCs from a healthy volunteer were stimulated with CMV-derived peptide and zoledronate. (B) PBMCs from a patient vaccinated with GPC3 peptide were stimulated with GPC3-derived peptide and zoledronate. The number indicates the number of cells. The presence of TCR-Vγ9^+^, CD3^+^ cells indicated γδ T cells. The presence of CD8^+^, dextramer^+^ cells indicated antigen-specific CTLs. HIV-dextramer was used as a negative control. (C) Analysis of the phenotype of CD8^+^, GPC3-dextramer^+^ cells at day 14. The CD45RA^−^, CCR7^−^ phenotype indicated the effector memory phenotype.

To assess whether this new culture method can induce the expansion of γδ T cells and peptide-specific CTLs, PBMCs were stimulated once with zoledronate and an antigen-derived peptide. Fig. 1A shows the representative data using PBMCs from a healthy volunteer. The number of total cells increased 3.2×10^2^-fold after 14 days (from 2.0×10^6^ to 6.4×10^8^). In flow cytometry analysis, γδ T cells increased 8.0×10^3^-fold after 14 days [from 5.6×10^4^ (2.8%) to 4.5×10^8^ (70%)]. Simultaneously with γδ T cells, CMV peptide-specific CTLs increased 1.9×10^4^-fold after 14 days [from 5.0×10^3^ (0.25%) to 9.6×10^7^ (15%)]. Similar results were obtained from three healthy subjects (data not shown).

Next, we investigated the capacity of this culture method to induce expansion of CTLs specific for peptides derived from the weakly immunogenic tumor-associated self-antigen GPC3. PBMCs from vaccinated patients were stimulated once with zoledronate and a GPC3-derived peptide. In Fig. 1B, the number of total cells increased 84-fold after 14 days (from 2.0×10^6^ to 1.7×10^8^). γδ T cells increased 4.0×10^3^-fold after 14 days [from 2.4×10^4^ (1.2%) to 9.5×10^7^ (56%)]. GPC3 peptide-specific CTLs increased 4.2×10^3^-fold after 14 days [from 4.4×10^3^ (0.22%) to 1.9×10^7^ (11%)]. In addition, during expansion, GPC3 peptide-specific CTLs acquired mainly an effector memory phenotype (CD45RA^−^, CCR7^−^) (Fig. 1C). One of the features of this culture method is the rate of increase in the number of cells. Table I shows the rate of increase in the number of cells in 16 patients with HCC. We found that the total cell number increased (range, 1.1–270-fold), γδ T cells increased (range, 23–1.1×10^4^-fold), and GPC3 peptide-specific CTLs increased (range, 24–1.7×10^5^-fold) after 14 days. These results suggest that peptide-specific CTLs were successfully expanded with the proliferation of γδ T cells from PBMCs by this new culture method.

### Efficiency of the culture method to induce expansion of GPC3 peptide-specific CTLs

**Figure 2:**
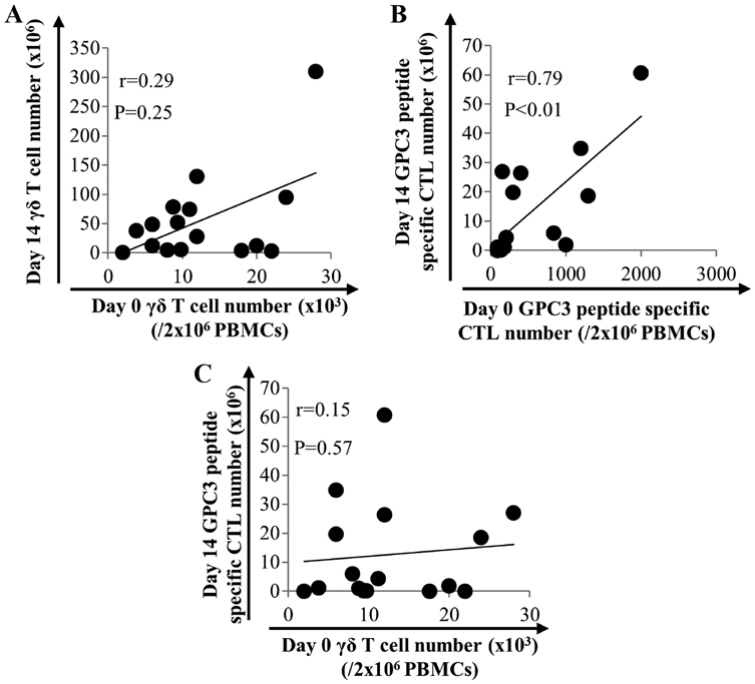
Efficacy of the method to induce expansion of GPC3 peptide-specific CTLs. (A) The correlation between the number of γδ T cells before and after expansion (n=16). (B) The correlation between the number of GPC3 peptide-specific CTLs before and after expansion. The number of GPC3 peptide-specific CTLs after expansion was correlated with that before expansion (n=16). (C) The correlation between the number of γδ T cells and the number of GPC3 peptide-specific CTLs after expansion (n=16).

One of the problems of cell transfer therapy is that it cannot predict cell growth prior to cell culture. Therefore, to identify predicting factors, we investigated the ability of this culture method to induce expansion of γδ T cells and GPC3 peptide-specific CTLs in 16 patients with HCC. As shown in Fig. 2, the number of γδ T cells after expansion did not correlate with that before expansion (Fig. 2A). On the other hand, the number of GPC3 peptide-specific CTLs after expansion correlated with that before expansion (P<0.01, r=0.79) (Fig. 2B). This result indicates that the number of GPC3 peptide-specific CTLs before expansion is a predicting factor. We expected a positive correlation between the number of γδ T cells and the number of GPC3 peptide-specific CTLs after expansion. However, no such correlation was observed (Fig. 2C).

### Activated γδ T cells function as antigen-presenting cells

**Figure 3:**
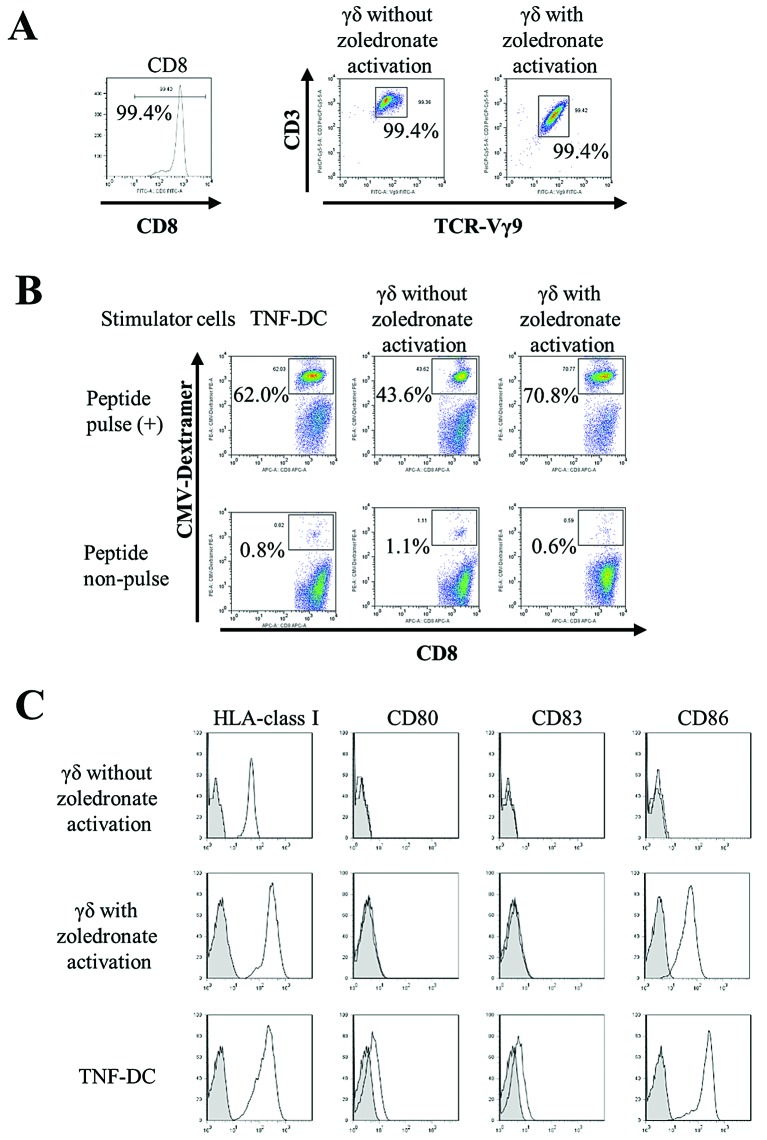
Activated γδ T cells function as antigen-presenting cells. (A) The percentages of sorted cells were analyzed using flow cytometry. The purity of sorted CD8^+^ cells, γδ T cells without zoledronate activation and γδ T cells with zoledronate activation were greater than 99%. (B) The responder CD8^+^ cells were co-cultured with stimulator cells pulsed with CMV peptide in the absence of zoledronate. After 2 weeks, flow cytometry analyses were performed using CMV-Dextramer. Non-pulsed stimulator cells were co-cultured with responder CD8^+^ as negative controls. Representative data are shown. Similar results were obtained from three healthy subjects. (C) Cell surface expression of antigen-presenting molecules (HLA-class I) and co-stimulatory molecules (CD80, CD83 and CD86) on γδ T cells (with or without zoledronate activation) and TNF-DCs using flow cytometry. Black line shows a specific antibody. Gray-filled area shows negative control. Representative data are shown. Similar results were obtained from three healthy subjects.

To examine whether the expansion of peptide-specific CTLs is enhanced by simultaneous activation/expansion of γδ T cells, we expanded peptide-specific CTLs in the absence of zoledronate. The purity of sorted CD8^+^ cells and γδ T cells with or without zoledronate activation was greater than 99% (Fig. 3A). The expansion of peptide-specific CTLs stimulated by γδ T cells with zoledronate activation (70.8%) was higher than by γδ T cells without zoledronate activation (43.6%). Moreover, the CTL-expanding ability of zoledronate-activated γδ T cells was comparable to that of TNF-DCs (62.0%), which are known professional antigen-presenting cells. These results indicate that zoledronate-activated γδ T cells function as antigen-presenting cells in co-cultures in the absence of zoledronate (Fig. 3B). We compared cell surface expression of antigen-presenting molecules and co-stimulatory molecules on γδ T cells (with or without zoledronate activation) and TNF-DCs. All cells expressed HLA-class I; however, γδ T cells without zoledronate activation did not express co-stimulatory molecules. Furthermore, CD86 expression in zoledronate-activated γδ T cells was comparable with that of TNF-DCs (Fig. 3C). These results indicate that γδ T cells activated by zoledronate acquire antigen-presenting properties accompanied by CD86 expression.

### Cytotoxic activity of expanded cells

**Figure 4:**
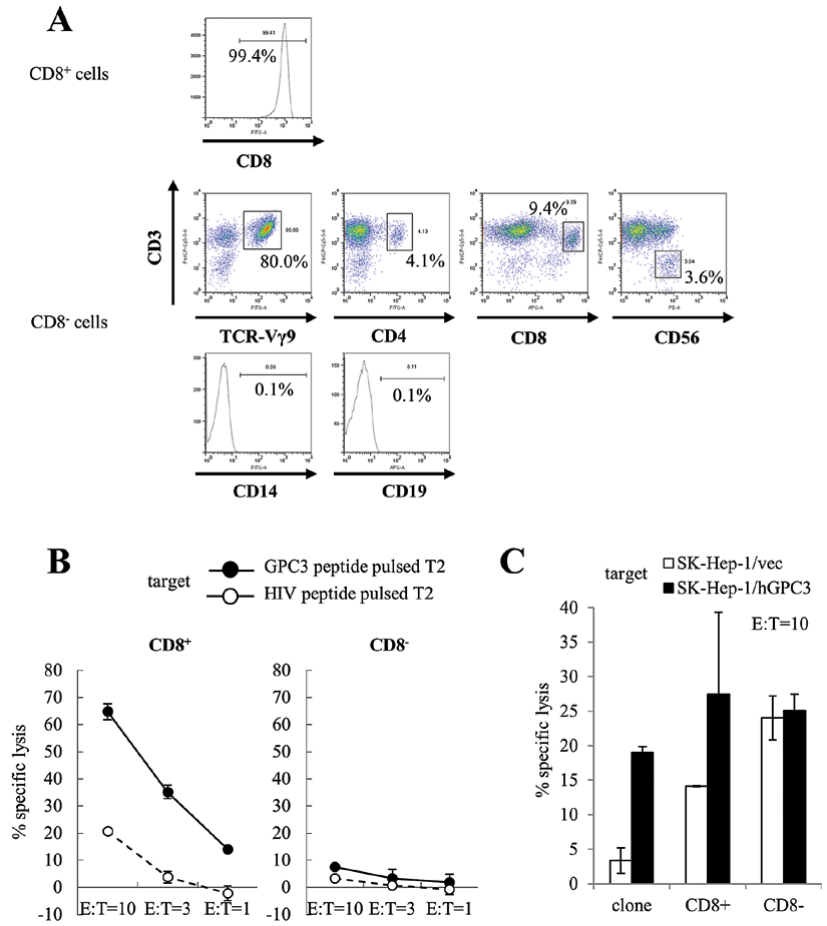
Cytotoxicity assay of cultured cells. We used CD8^+^ and CD8^−^ cells that were isolated from cultured cells using CD8 microbeads at day 14 as effector cells. A GPC3 peptide-specific CTL clone was used as a positive control. We performed cytotoxicity assays using expanded cells from four patients. Similar results were obtained in three of the four patients. Representative data are shown. (A) We examined the purity of the CD8^+^ cell populations obtained for these experiments. We performed further immunophenotyping of CD8^−^ cells using flow cytometry. (B) T2 cells pulsed with GPC3 (black circle) or HIV (white circle) peptide were used as target cells. CD8^+^ cells (left) showed GPC3 peptide-specific cytotoxic activity. CD8^−^ cells (right) did not show GPC3 peptide-specific cytotoxic activity. (C) SK-Hep-1/hGPC3 (black bar) or SK-Hep-1/vec (white bar) cells were used as target cells. CD8^+^ cells showed GPC3-specific cytotoxicity, whereas CD8^−^ cells showed cytotoxicity against SK-Hep-1 cells, but not GPC3 specificity (E:T=10). Data represent the means ± SD.

We performed a cytotoxicity assay to assess the peptide specificity and cytotoxic activity of expanded cells against cancer cells. We used CD8^+^ and CD8^−^ cells that were isolated from cultured cells using CD8 microbeads at day 14 as effector cells. The purity of CD8^+^ cells was 99.4%. We performed further immunophenotyping of CD8^−^ cells. CD3^+^ Vg9^+^ cells were 80.0% of CD8^−^ cells. CD8^−^ cells also included CD3^+^ CD4^+^ cells (4.1%), CD3^+^ CD8^+^ cells (9.4%), and CD3^−^ CD56^+^ cells (NK cells; 3.6%). CD14^+^ cells (monocytes; 0.1%) and CD19^+^ cells (B cells; 0.1%) were not observed in CD8^−^ cells. These results indicate that CD8^−^ cells were predominantly γδ T cells (Fig. 4A). Similar results were obtained from four patients. CD8^+^ cells showed cytotoxicity against T2 cells pulsed with GPC3 peptide, whereas CD8^−^ cells did not show cytotoxicity against T2 cells pulsed with both GPC3 and HIV peptide (Fig. 4B). Moreover, we used SK-Hep-1/hGPC3 cells as target cells; they were transfected with the GPC3 gene and endogenously presented GPC3 peptide. CD8^+^ cells showed GPC3-specific cytotoxicity, whereas CD8^−^ cells showed cytotoxicity against SK-Hep-1 cells but did not show GPC3 specificity (Fig. 4C). We performed cytotoxicity assays using expanded cells from four patients. Similar results were obtained in three of the four patients. These results indicate that CD8^+^ cells included mostly GPC3 peptide-specific CTLs that had cytotoxic activity against cancer cells and endogenously presented GPC3 peptide, and CD8^−^ cells included mostly γδ T cells that had cytotoxic activity against cancer cells.

### Antitumor activity of γδ T cells and GPC3-specific CTLs in vivo

**Figure 5:**
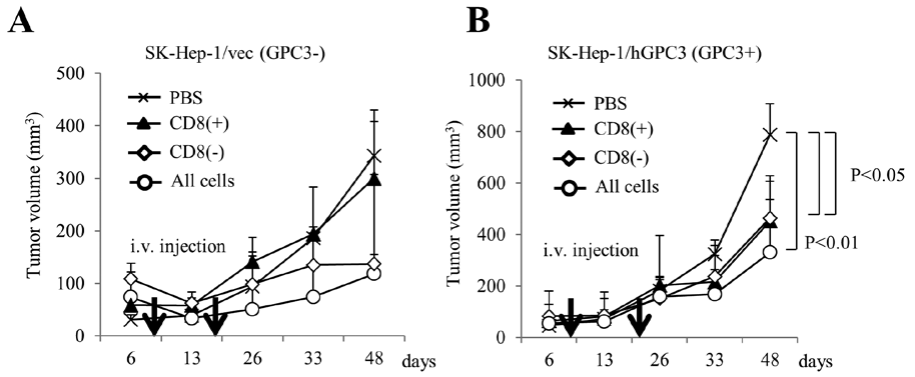
Antitumor activity of γδ T cells and GPC3-specific CTLs *in vivo*. We subcutaneously inoculated (A) SK-Hep-1/vec or (B) SK-Hep-1/hGPC3 cells into NOD/SCID mice and intravenously injected effector cells. We performed adoptive cell transfer of expanded cells using five mice per group. Data represent the means ± SD. (A) The growth of SK-Hep-1/vec treated with CD8^−^ cells or all cells was significantly inhibited. The growth of SK-Hep-1/vec treated with CD8^+^ cells was not inhibited. (B) The growth of SK-Hep-1/hGPC3 treated with CD8^+^ or CD8^−^ cells was significantly inhibited. In addition, treatment with all cells, including both CD8^+^ and CD8^−^ cells, showed an additive inhibitory effect.

We performed adoptive cell transfer of expanded cells in a mouse model. We subcutaneously inoculated SK-Hep-1/vec (Fig. 5A) or SK-Hep-1/hGPC3 (Fig. 5B) cell lines into NOD/SCID mice and intravenously injected effector cells twice. As effector cells, we used CD8^+^ or CD8^−^ cells that were isolated from cultured cells using CD8 microbeads at day 14, and we used all cells that included both CD8^+^ and CD8^−^ cells. As shown Fig. 5B, the growth of SK-Hep-1/hGPC3 treated with CD8^+^ or CD8^−^ cells was significantly inhibited compared with the negative control. In addition, treatment of all cells, including both CD8^+^ and CD8^−^ cells, tended to show an additive inhibitory effect. On the other hand, the growth of SK-Hep-1/vec that was inhibited in the treatment of CD8^−^ or all cells was not inhibited by treatment of CD8^+^ cells (Fig. 5A). These results indicate that cultured cells had antitumor effects due to the respective CD8^+^ and CD8^−^ cells.

## Discussion

Specific cellular immunotherapy of cancer requires efficient generation and expansion of CTLs that recognize tumor-associated antigens. ACT with TILs isolated from metastatic melanoma lesions lead to objective tumor regression. However, TILs can be exploited only in melanoma patients with resectable tumors and from which T cells can be expanded *ex vivo*. An alternative approach has been explored for patients with other types of tumor using autologous lymphocytes isolated from peripheral blood. Various clinical trials involving adoptively transferred autologous T cells transduced with a TCR or chimeric antigen receptors have been conducted (35–37). Clinical trials using our culture method should be performed in the future.

The standard approach to generating tumor-specific CTLs is based on antigen presentation by dendritic cells (DCs). Although DCs are the most efficient APCs known so far, serious drawbacks to their use in adoptive immunotherapy exist, including their scarcity in the peripheral blood, their limited expansion and their functional heterogeneity. These limitations have motivated an intense search for alternative sources of APCs. An antigen-presenting function of γδ T cells was suggested by recent observations that upon activation, these cells acquire phenotypic and functional characteristics of professional APCs concomitant with the capacity to induce primary CD4^+^ and CD8^+^ T-cell responses to antigens (30–33). To the best of our knowledge, this is the first report of the simultaneous expansion of γδ T cells and antigen-specific CTLs from the PBMCs of patients.

Most adoptive CTL transfer studies in patients with tumors used approximately 10^8^ to 10^11^ T cells/m^2^ body surface area of the patient (38). The expansion of CTLs from PBMCs of vaccinated patients with advanced HCC yields cell numbers sufficient for adoptive transfer. Theoretically, the number of GPC3-specific CTLs obtained for apheresis (10 L) is, at most, 1.5×10^11^ cells.

One reason for the scarcity of adoptive immunotherapy is the individual variability in cell growth. *In vitro*, it is difficult to adequately expand antigen-specific CTLs in most patients with cancer. In addition, cell growth cannot be predicted before culture. Therefore, to identify predicting factors, we investigated the efficacy of this culture method in inducing expansion of GPC3 peptide-specific CTLs in 16 patients with HCC. The prediction of cell growth may enable the implementation of personalized medicine.

In this study, we assessed the expansion of GPC3 peptide-specific CTLs using PBMCs from vaccinated patients with HCC. GPC3 is also overexpressed in other malignant tumors, such as melanoma, Wilms’ tumor, hepatoblastoma, yolk sac tumor, ovarian CCC and lung squamous cell carcinoma (39–43). Adoptive transfer of GPC3 peptide-specific CTLs may also be available for other GPC3-expressing cancers.

This culture method has a limitation. We performed this culture using PBMCs of the same person both before and after vaccination. GPC3 peptide-specific CTLs could be induced from the PBMCs of all patients after vaccinations. However, this method failed to induce GPC3 peptide-specific CTLs from the PBMCs of patients before vaccination. Similarly, GPC3 peptide-specific CTLs could not be induced from the PBMCs of healthy donors (data not shown). These results may have been caused by the low frequency of cancer antigen-specific CTLs in peripheral blood before vaccination. These results suggest that to increase GPC3 peptide-specific CTLs, vaccination is effective before cell culture. On the other hand, with regard to CMV-derived peptide, CMV peptide-specific CTLs could be induced with the proliferation of γδ T cells from the PBMCs of healthy donors by this culture method. This culture method may also be available for other antigens.

Adoptive cell transfer of all cells after expansion, including both CTLs and γδ T cells, significantly inhibited tumor growth in a mouse model. Tumor cells acquire various immune escape mechanisms including loss of antigens or the HLA-class I molecule. It may be effective to use both CTLs and γδ T cells because they have different antigen recognition abilities. However, we did not confirm that the results were due to either synergy or an additive effect. Because activated γδ T cells produce large amounts of interferon-γ, it may be a synergy effect. Analysis of the mechanisms of the effectiveness of CTLs and γδ T cells is a future challenge.

On the other hand, we previously reported that intratumor peptide injection was an effective method of enhancing tumor cell antigenicity and that it showed an induced antigen-spreading effect *in vivo* (44,45). Moreover, we are investigating the antitumor activity of γδ T cells against HCC cells pretreated with zoledronate. The combination of these pretreatments that enhance tumor cell antigenicity and adoptive immunotherapy using CTLs and γδ T cells may be a useful application for cancer therapy.

In conclusion, this study indicates that simultaneous expansion of γδ T cells and peptide-specific CTLs using zoledronate are useful for adoptive immunotherapy.

## Figures and Tables

**Table I:** Rate of increase in the number of cells in 16 patients with HCC.

		Total			γδ			GPC3 specific CTLs		
				
Sample	HLA-A	Day 0	Day 14	The rate of increase	Day 0	Day 14	The rate of increase	Day 0^a^	Day 14^b^	The rate of increase
1	02:01	2.0×10^6^	1.7×10^8^	85	1.2×10^4^	2.8×10^7^	2.3×10^3^	2.0×10^3^	6.1×10^7^	3.1×10^4^
2	02:01	2.0×10^6^	5.4×10^8^	2.7×10^2^	2.8×10^4^	3.1×10^8^	1.1×10^4^	1.6×10^2^	2.7×10^7^	1.7×10^5^
3	02:01	2.0×10^6^	1.7×10^8^	85	8.8×10^3^	7.8×10^7^	8.9×10^3^	92	1.0×10^6^	1.1×10^4^
4	02:01	2.0×10^6^	1.7×10^8^	85	2.4×10^4^	9.5×10^7^	4.0×10^3^	1.3×10^3^	1.9×10^7^	1.5×10^4^
5	02:01	2.0×10^6^	7.4×10^7^	37	9.4×10^3^	5.2×10^7^	5.5×10^3^	92	1.8×10^5^	2.0×10^3^
6	02:01	2.0×10^6^	2.5×10^7^	13	2.0×10^4^	1.2×10^7^	6.0×10^2^	1.0×10^3^	1.9×10^6^	1.9×10^3^
7	02:01	2.0×10^6^	1.6×10^8^	80	1.1×10^4^	7.4×10^7^	6.7×10^3^	2.1×10^2^	4.4×10^6^	2.1×10^4^
8	02:01	2.0×10^6^	4.0×10^8^	2.0×10^2^	1.2×10^4^	1.3×10^8^	1.1×10^4^	4.0×10^2^	2.6×10^7^	6.5×10^4^
9	02:01	2.0×10^6^	4.0×10^7^	20	8.0×10^3^	4.4×10^6^	5.5×10^2^	8.4×10^2^	5.9×10^6^	7.0×10^3^
10	02:01	2.0×10^6^	2.2×10^6^	1.1	2.0×10^3^	4.5×10^4^	23	92	2.2×10^3^	24
11	02:01	2.0×10^6^	1.5×10^8^	75	6.0×10^3^	4.8×10^7^	8.0×10^3^	3.0×10^2^	2.0×10^7^	6.7×10^4^
12	02:01	2.0×10^6^	1.1×10^8^	55	6.0×10^3^	1.2×10^7^	2.0×10^3^	1.2×10^3^	3.5×10^7^	2.9×10^4^
13	24:02	2.0×10^6^	5.8×10^6^	2.9	1.8×10^4^	3.8×10^6^	2.1×10^2^	1.3×10^2^	6.3×10^4^	4.9×10^2^
14	24:02	2.0×10^6^	4.0×10^6^	2	2.2×10^4^	2.6×10^6^	1.2×10^2^	1.0×10^2^	3.1×10^4^	3.1×10^2^
15	24:02	2.0×10^6^	9.9×10^7^	50	3.8×10^3^	3.8×10^7^	1.0×10^4^	1.8×10^2^	1.2×10^6^	6.7×10^3^
16	24:02	2.0×10^6^	4.0×10^7^	20	9.8×10^3^	5.6×10^6^	5.7×10^2^	1.4×10^2^	1.4×10^5^	1.0×10^3^

a Frequency of GPC3-specific CTLs of 2×10^6^ PBMCs was measured by *ex vivo* IFN-γ ELISPOT assay.

b GPC3-specific CTLs after cell culture were measured by flow cytometry.
